# *Aureobasidium melanigenum* catheter-related bloodstream infection: a case report

**DOI:** 10.1186/s12879-022-07310-9

**Published:** 2022-04-05

**Authors:** Shinya Yamamoto, Mahoko Ikeda, Yuki Ohama, Tomohiro Sunouchi, Yasutaka Hoshino, Hiroshi Ito, Marie Yamashita, Yoshiaki Kanno, Koh Okamoto, Satoshi Yamagoe, Yoshitsugu Miyazaki, Shu Okugawa, Jun Fujishiro, Kyoji Moriya

**Affiliations:** 1 Department of Infectious Disease, The University of Tokyo Hospital, 7-3-1, Hongo, Bunkyo-ku, Tokyo, 113-8655 Japan; 2 Department of Infection Control and Prevention, The University of Tokyo Hospital, 7-3-1, Hongo, Bunkyo-ku, Tokyo, 113-8655 Japan; 3 Department of Pediatric Surgery, The University of Tokyo Hospital, 7-3-1, Hongo, Bunkyo-ku, Tokyo, 113-8655 Japan; 4 Department of Chemotherapy and Mycoses, National Institute of Infectious Diseases, Toyama 1-23-1, Shinjuku-ku, Tokyo, 162-8640 Japan

**Keywords:** *Aureobasidium melanigenum*, Catheter-related bloodstream infection, DNA sequence-based identification, Dimorphic fungus

## Abstract

**Background:**

*Aureobasidium melanigenum* is a ubiquitous dematiaceous fungus that rarely causes invasive human infections. Here, we present a case of *Aureobasidium melanigenum* bloodstream infection in a 20-year-old man with long-term catheter use.

**Case presentation:**

A 20-year-old man receiving home care with severe disabilities due to cerebral palsy and short bowel syndrome, resulting in long-term central venous catheter use, was referred to our hospital with a fever. After the detection of yeast-like cells in blood cultures on day 3, antifungal therapy was initiated. Two identification tests performed at a clinical microbiological laboratory showed different identification results: *Aureobasidium pullulans* from matrix-assisted laser desorption/ionization time-of-flight mass spectrometry, and *Cryptococcus albidus* from a VITEK2 system. Therefore, we changed the antifungal drug to liposomal amphotericin B. The fungus was identified as *A. melanigenum* by DNA sequence-based analysis. The patient recovered with antifungal therapy and long-term catheter removal.

**Conclusion:**

It is difficult to correctly identify *A. melanigenum* by routine microbiological testing. Clinicians must pay attention to the process of identification of yeast-like cells and retain *A. melanigenum* in cases of refractory fungal infection.

## Background

*Aureobasidium* is a ubiquitous, saprophytic, dematiaceous fungus found in fresh water, oil-treated wood, and even in houses [1–3]. It produces various useful materials, such as pullulan [4], which is used as a biocontrol product against post-harvest diseases of fruits and vegetables [5], and cosmetic and biomedical applications, such as drug delivery, gene targeting, tissue engineering, and vaccination [6]. Therefore, research on its bioproducts is a hot topic in several fields, including biotechnology [7] and biomedical research [8].

*Aureobasidium* species are also known to be causative pathogens of human infections, especially *A. pullulans. A. pullulans* subspecies has been redefined to four species (*A. pullulans, A. melanigenum, A. subglaciale,* and *A. nambiae*) since 2014 [1]. The virulence of *Aureobasidium* species in a murine sepsis model showed no significant difference among species; however, *A. melanigenum* with darkly pigmented colonies showed greater lethality [9].

In humans, *Aureobasidium* infection is usually cutaneous because it occurs due to traumatic inoculation of the skin [10–12] and eye [13, 14]. However, bloodstream infection due to an indwelling catheter can also occur [9, 15–17]. Data on *Aureobasidium* infection in humans are limited, especially regarding its diagnosis and management. Herein, we describe a case of *A. melanigenum* catheter-related bloodstream infection (CRBSI) in a patient receiving home care.

## Case presentation

A 20-year-old man receiving home care with a long-term central venous catheter (CVC) was referred to our hospital with fever and dyspnea (day 0). Central parenteral nutrition through the CVC was started 2 years prior. His medical history included severe neurological disabilities due to cerebral injury after influenza encephalopathy, cardioplasty, esophagogastric disconnection, and small bowel resection due to postoperative ileus 3 years ago. His medications included lansoprazole, carbamazepine, triclofos sodium, baclofen, and daikenchuto.

On admission, he had a high-grade fever of 38.6 °C and chills. Physical examination revealed no significant abnormalities, except for scattered erythema. Laboratory data were as follows: white blood cell count 5700/μL; hemoglobin 11.8 g /dL; platelet count 243,000/μL; albumin 3.5 g/dL; total bilirubin 0.3 mg/dL; aspartate aminotransferase 20 U/L; alanine aminotransferase 33 U/L; γ-glutamyl transpeptidase, 178 U/L; serum creatinine 0.44 mg/dL; C-reactive protein 4.97 mg/dL; and procalcitonin 0.30 ng/mL. Cefazolin was started at a dosage of 0.5 g every 12 h due to a suspected CRBSI following collection of one set of blood cultures from the CVC. He still had fever between 38.1 and 39.3 °C and chills during cefazolin administration.

**Fig. 1:**
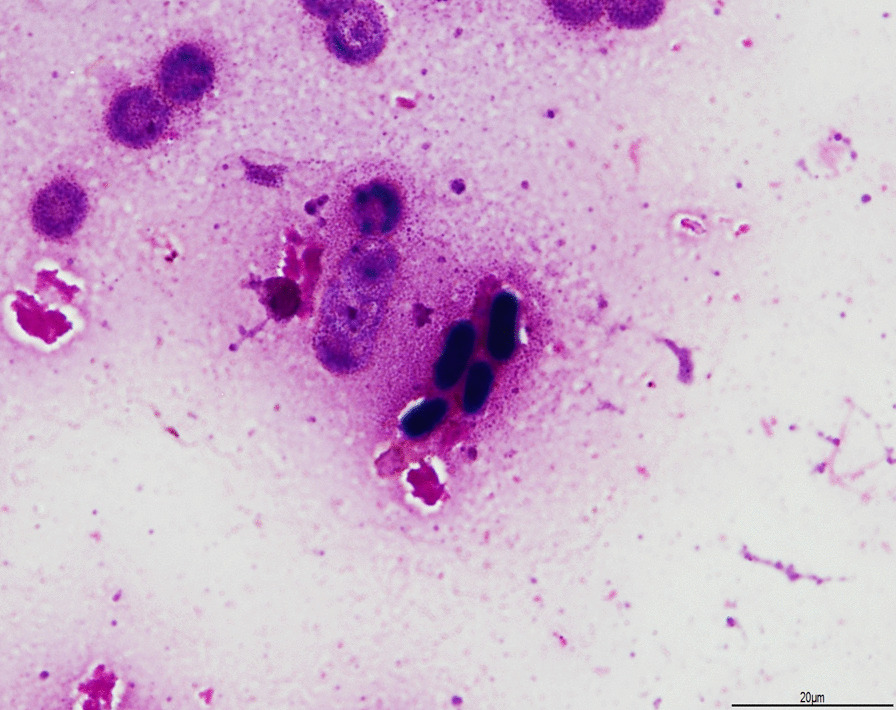
Microscopy images of *Aureobasidium melanigenum* Gram stain (1000 ×) of positive blood cultures

On day 3, yeast-like cells grew in an aerobic blood culture bottle obtained on admission (Fig. 1a). The serum beta-D glucan measured was 10.8 pg/mL (Wako Pure Chemical Industries, Ltd., Tokyo, Japan; cut-off value, < 11 pg/mL). Retinal examination revealed no evidence of endophthalmitis. Micafungin was added at a dose of 100 mg every 24 h against suspected candidemia after an additional set of blood cultures, and the CVC was removed. The patient became afebrile after initiating systemic antifungal therapy. The colony appeared rough and pale orange in color. The colony’s India ink stain did not show a zone of clearance of ink particles, like *Cryptococcus*. Colonies were tested on CHROMagar Candida medium (Kanto Chemical Co., Inc., Tokyo, Japan) using matrix-assisted laser desorption/ionization time-of-flight mass spectrometry (MALDI-TOF MS, the MALDI Biotyper Reference Library Ver.4.0.0.1, Filamentous Fungi Library Ver.1.0; Bruker Daltoniks, Germany), identifying them as *Aureobasidium pullulans* (score 1.919). On day 6, the high fever recurred. Micafungin was stopped and liposomal-amphotericin B (L-AMB) was started (3 mg/kg/day) for *Cryptococcus albidus* owing to a 90% probability of identification using the VITEK2 COMPACT Microbial Detection System (version 8.01 database: SYSMEX bioMerieux Co., Ltd., Tokyo, Japan).

The treatment was subsequently stopped because urticaria appeared after the 3-day use of L-AMB. After the discontinuation of L-AMB, no additional systemic antibiotics were administered. The patient remained afebrile. Minimum inhibitory concentrations (MIC) according to the reference method for broth dilution antifungal susceptibility testing of yeasts [18] were as follows: micafungin 0.25 μg/mL; caspofungin 0.5 μg/mL; AMPH-B 1 μg/mL; 5-flucytosine 0.25 μg/mL; fluconazole 8 μg/mL; itraconazole 0.06 μg/mL; and voriconazole 0.03 μg/mL.

The fungus was identified as *Aureobasidium*; however, it was impossible to differentiate between *A. pullulans* and *A. melanigenum* by sequencing the internal transcribed spacer regions. Comparison of the sequence from the isolate to sequences in the MYCOBANK database (https://www.mycobank.org/) revealed 99.82% similarity of the sequence with *A. pullulans* CBS 110.67 strain and 99.83% similarity with *A. melanigenum* CBS 123.37 strain. Next, the other two regions, β-tubulin and elongase, were compared using the BLAST database (http://www.ncbi.nlm.nih.gov/BLAST). The sequence of the isolate showed 88.5% similarity with *A. pullulans* CBS 584.75 strain and 94.20% similarity with *A. melanigenum* CBS 105.22 strain in the β-tubulin gene region respectively, and in the elongase gene region, the sequence of the isolate showed 92.37% similarity with *A. pullulans* CBS 584.75 strain and 95.22% similarity with *A. melanigenum* CBS 109800 strain.

**Fig. 2:**
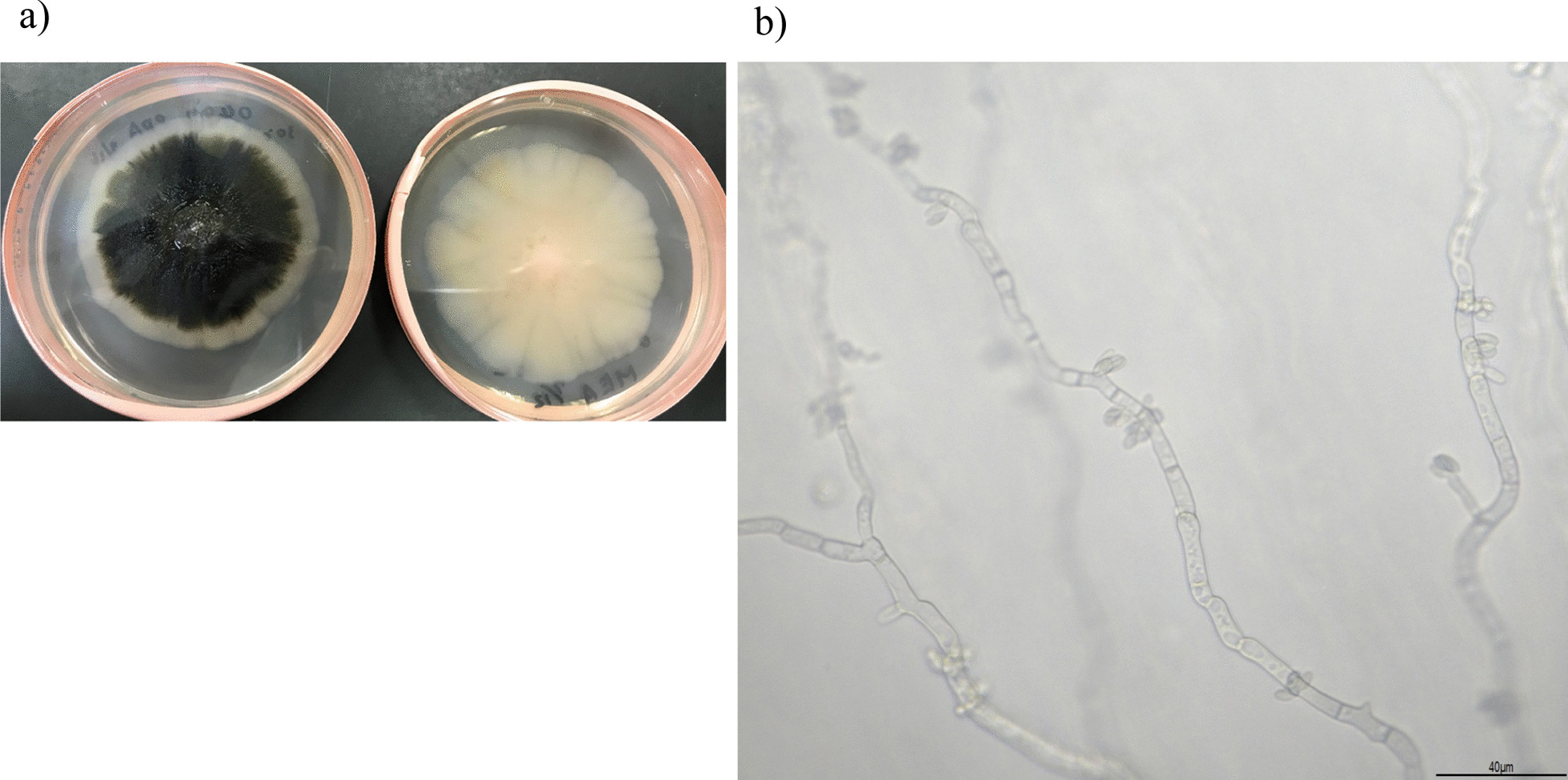
Colonies and microscopy images of *Aureobasidium melanigenum* **a** Colonies of *Aureobasidium melanigenum* cultured on potato dextrose agar medium (left) and on malt extract agar medium (right) at 30 °C for 15 days. **b** Conidiogenous cells and filament hypha without staining incubated on potato dextrose agar with the slide culture method for 3 days at 30 °C

Therefore, the fungus was identified based on clustering in the phylogenetic analysis of β-tubulin and elongase genes with representative strains [19]. A phylogenetic tree was constructed by the neighbor-joining method using the MEGA X program [20] from the distance data generated by multi-alignment of the nucleotide sequence. A bootstrap value of 70% or more was considered as moderate support for the identification of clades. This fungus was placed in *the A. melanogenum* clade in the phylogenetic tree. Thus, the fungus was identified as *A. melanogenum*. The colonies were black (Fig. 2a), and conidiogenous cells were also detected (Fig. 2b). Fungemia was dismissed after day 7. The patient was discharged on day 27 without complications. There was no recurrence of *A. melanigenum* bloodstream infection.

## Discussion and conclusions

We describe a case of CRBSI caused by *A. melanigenum*, which was successfully treated by removing the CVC and antifungal therapy. The identification of *Aureobasidium* species using routine microbiological tests is difficult.

Little is known about the predisposing factors for *Aureobasidium* infections. Previous case reports and studies revealed the risk factors of *Aureobasidium* infection, such as immunosuppressant use, steroids and HIV infection [21], cancer (with chemotherapy) [22], transplantation [23], trauma history [24], asplenia [25], leukemia [25, 26], and parenteral nutrition [21, 22]. *Aureobasidium* might be a more important pathogen in the future because of iatrogenic immunosuppression, such as solid organ transplant and massive chemotherapy, and artificial devices, such as central venous ports, would be increasing. In this case, the patient was not immunodeficient but might have been malnourished due to prolonged intravenous nutrition. The relationship between the medication used in this case and *Aureobasidium* infection was unclear.

The identification of *A. melanigenum* infection is difficult before the colonies darken. We could not confirm the species in the early phase of the case; hence, we could not suspect *Aureobasidium* infection. In addition, *A. melanigenum* is a dimorphic cell that initially mimics *Candida* species. The patient was initially diagnosed with candidemia based on the results of Gram staining. Conventional identification using MALDI-TOF MS or VITEK2 systems might also have the risk of misidentification, depending on the version of the database and the method of identification (e.g., in this case, MALDI-TOF MS database had only *A. pullulans* in *Auerobasidium* species). VITEK2 and MALDI-TOF MS analyses yielded different results for the identification of fungi, and both were incorrect in this case. Molecular methods play an important role in confirming the identity of this organism. Because the sequencing of internal transcribed spacer regions could not differentiate between *A. pullulans* and *A. melanigenum*, additional sequencing of different regions, such as the β–tubulin gene and elongase gene, might be needed. Clinicians should consider that yeast-like cells suggest not only *Candida* and *Cryptococcus* species but also other pathogens, including *A. melanigenum,* although *Auerobasidium* remains a rare causative organism of catheter-related infections.

There are no established criteria for antifungal susceptibility of *Aureobasidium* species. *A. melanigenum* and *A. pullulans* showed similar antifungal susceptibility patterns, such as low MICs of amphotericin B, itraconazole, and posaconazole, and both had high MICs for fluconazole [23]. These susceptibilities were reported in a previous clinical report [24], and the pattern was the same as that observed in our case. Amphotericin B is recommended for *Aureobasidium* spp. in the clinical guidelines for systemic phaeohyphomycosis [25], although there is no standardized treatment for *Aureobasidium* infection.

For non-pharmacological management, catheter removal is the most important treatment approach for CRBSI caused by *A. melanigenum*. A previous report indicated that catheter removal was necessary for CVC-related *A. pullulans* septicemia [17]. Removal of the CVC might be critical to resolve fungemia because in our case, systemic antifungal therapy could not be continued for a sufficient duration. Hand hygiene before CVC care at home is also important to prevent infection by such fungi [26].

We have highlighted a case of CRBSI caused by *A. melanigenum*. Identification of *A. melanigenum* is difficult using conventional diagnostic approaches, and molecular methods play an important role in confirming *A. melanigenum* infection. Further studies are needed to determine the appropriate treatment for *Auerobasidium* infections.

## Data Availability

Not applicable.

## Abbreviations

CRBSI: Catheter-related bloodstream infection
CVC: Central venous catheter
MALDI-TOF MS: Matrix-assisted laser desorption/ionization time-of-flight mass spectrometry
L-AMB: Liposomal-amphotericin B
MIC: Minimum inhibitory concentrations
